# Technological Implications of Modifying the Extent of Cell Wall-Proanthocyanidin Interactions Using Enzymes

**DOI:** 10.3390/ijms17010123

**Published:** 2016-01-18

**Authors:** Ana Belén Bautista-Ortín, Rim Ben Abdallah, Liliana del Rocío Castro-López, María Dolores Jiménez-Martínez, Encarna Gómez-Plaza

**Affiliations:** 1 Department of Food Science and Technology, Faculty of Veterinary, University of Murcia, 30071 Murcia, Spain; anabel@um.es (A.B.B.-O.); mariadolores.jimenezm@um.es (M.D.J.-M.); 2 Université de Tunis El Manar, Faculté des Sciences de Tunis, 2092 Tunis, Tunisia; rima_bio@hotmail.com; 3 LIP-MB, National Institute of Applied Sciences and Technology (INSAT), University of Carthage, 2046 Tunis, Tunisia; 4 Departamento de Ingeniería Química, Universidad de las Américas Puebla, Alimentos y Ambiental, Sta. Catarina Mártir, 72810 San Andrés Cholula, Puebla, México; lilianar.castrolz@udlap.mx

**Keywords:** proanthocyanidin, tannins, cell wall, enzymes, polysaccharides, phloroglucionolysis, size exclusion chromatography, polygalacturonase, cellulase

## Abstract

The transference and reactivity of proanthocyanidins is an important issue that affects the technological processing of some fruits, such as grapes and apples. These processes are affected by proanthocyanidins bound to cell wall polysaccharides, which are present in high concentrations during the processing of the fruits. Therefore, the effective extraction of proanthocyanidins from fruits to their juices or derived products will depend on the ability to manage these associations, and, in this respect, enzymes that degrade these polysaccharides could play an important role. The main objective of this work was to test the role of pure hydrolytic enzymes (polygalacturonase and cellulose) and a commercial enzyme containing these two activities on the extent of proanthocyanidin-cell wall interactions. The results showed that the modification promoted by enzymes reduced the amount of proanthocyanidins adsorbed to cell walls since they contributed to the degradation and release of the cell wall polysaccharides, which diffused into the model solution. Some of these released polysaccharides also presented some reactivity towards the proanthocyanidins present in a model solution.

## 1. Introduction

The existence of interactions between proanthocyanidins (PAs) and cell wall material (CW), more precisely with the polysaccharides found therein, has been demonstrated by several research groups [1,2,3]. However, although less studied, anthocyanins also present an affinity for cell walls [4,5] and such interactions could limit their content in foods where these chemical compounds play an important organoleptic role. These interactions may also have technological implications in some food industries, such as winemaking [6,7], apple juice manufacturing [8,9], and pear canning [10]. They may, for example, explain the low quantities of proanthocyanidins that are found in musts and juices compared with the initial quantities measured in the fruits.

In the above industries, maceration enzymes are used for deconstructing the polysaccharide network of the plant cell walls by partial hydrolysis of the structural polysaccharides (mainly pectins, hemicelluloses, and cellulose), facilitating the extraction of the phenolic compounds located inside the vacuoles [11,12]. The commercial enzymes used in food industry are complex mixtures of pectolytic enzymes (which mainly contain polygalacturonase, and to a lesser extent, pectin methyl esterase and pectin lyase activities), cellulases, hemicellulases, and acid proteases enzymes [11].

Our research group carried out preliminary studies of the effect of a commercial maceration enzyme on the complexes that PAs and cell walls (CWs) form, to determine whether they may limit or favor interactions and/or whether they participate in the desorption processes of these compounds from cell walls once they are adsorbed [13]. The results showed that cell wall polysaccharides adsorbed a high amount of proanthocyanidins, and that the presence of enzymes in the solution reduced proanthocyanidin-cell wall interactions, probably through the elimination of pectins from the cell wall network. These findings may be of technological interest since the use of maceration enzymes will favor an increase in the proanthocyanidin content of must and juices, not only by favoring the extraction of these compounds from skin cell vacuoles but also by decreasing their adsorption to CWs [13].

Moreover, in enology, there is another possible technological interest arising from a deeper knowledge of PA–CW interactions; that is, using the pomace obtained after maceration as a fining agent to reduce the level of proanthocyanins in wines when they show a high degree of astringency [14,15,16]. Pomace wastes from the winemaking are widely available and a source of cell wall material. How enzymes may be used to enhance or limit, if necessary, the CW adsorption properties would be of great interest. Our previous study confirmed that pomace cell wall material showed a very high degree of affinity for proanthocyanidins, similar to the retention exerted by fresh grape cell walls, meaning that these pomace cell walls could be used in wines to reduce astringency. Furthermore, first results suggest that the use of maceration enzymes could affect the retention capacity of the pomace cell walls and the extent to which high or low molecular mass proanthocyanidins are retained, which might have implications for the sensory properties of wine.

Therefore, it is very important to deeper our knowledge on the nature of these interactions and the role played by the main enzymatic activities found in the commercial enzymes in the ability to disrupt or manage these associations, as well as the effect of the polysaccharides released after the action of these enzymes on the quantities of PAs in solution.

## 2. Results and Discussion

Cell walls adsorb proanthocyanidins and other phenolics due to their chemical composition. Cell walls are composed of about 90% polysaccharides and 10% structural proteins. The polysaccharides are generally grouped into three categories: cellulose, pectins, and hemicelluloses. Hemicellulose cross-link cellulose microfibrills strengthen cell walls and are also embedded in pectic polysaccharides [17]. Pectins are galacturonate-rich acidic polysaccharides and represent up to 30% of the cell walls. The different structural classes of pectic polysaccharides found in plant cells are homogalacturonan and ramnogalacturonan II and I. Homogalaturonan is composed of galacturonic acid (GalA) residues linked by β-1,4-bond and which can be methyl-esterified at the C-6 carboxyl and/or acetylated at the O-2 or O-3. The backbone of homogalacturonan is covalently linked to ramnogalacturonan-I, ramnogalacturonan-II and presumably covalently cross-linked to hemicellusose [18].

Two experiments were conducted in this study. The first one consisted on suspending the purified grape skin CWs in a model solution containing the PA or the PA and one of the following enzymes: polygalacturonase (PG), cellulase (CEL), and a commercial maceration enzyme (CE). The purified enzyme preparations were used in single hydrolysis reactions to evaluate their contribution to the degradation of skin cell walls. The commercial enzyme used in the experiments was chosen since it is designed for degrading different polysaccharide targets [19]. This experiment will help to determine how the different enzymes affect the interaction of PA with CWs and if the commercial enzyme, containing as main activities this two activities together made any difference.

The extent of CWM-PA interaction, both in the control samples (those with no added enzyme) and in the samples with enzyme, was determined by observing the concentration of the PAs that remained in solution after the interaction (Table 1).

**Table 1:** Concentration and composition of the remaining PAs in solution after the interaction with CW in the presence or absence of enzymes.

Sample	Total mg/L	%Retention *	mDP	%Galloylation
PA	853.47 ± 60.92a		2.36 ± 0.01a	11.15 ± 0.02a
CW + PA	365.96 ± 43.80d	57.2	1.81 ± 0.05d	8.30 ± 0.33d
CW + PG + PA	543.89 ± 40.50c	36.4	1.90 ± 0.10e	8.70 ± 0.06c
CW + CEL + PA	619.19 ± 15.02b	27.5	2.07 ± 0.06b	9.76 ± 0.05b
CW + CE + PA	576.77 ± 40.98bc	32.5	2.04 ± 0.01c	8.23 ± 0.05d

* Calculated as the difference between PA concentration in the solution before and after the interaction with CWs. mDP: mean degree of polymerization; CW + PA represents the control samples, without any enzyme addition, as explained in the Material and Methods section; PG: polygalacturonase; CEL: cellulase; CE: commercial enzyme. Different letters within the same column indicate significant differences (*p* < 0.05) according to a Least Significant Difference test.

Maximum PA retention was observed when no enzyme was present in the model solution, since the PAs remaining in this model solution presented the lowest values (a concentration of 365.96 mg/L of proanthocyanidins remaining in the solution, indicating a retention of 57.2%). The presence of the different enzymes in the solution lowered the retention of the PA in the CW in all cases (from 57.2% to a minimum value of 27.5% when cellulase was used). The major effect of these enzymes was the loosening of the cell wall structure and a depectination (and pectins have been described as the polysaccharides presenting the highest affinity for PAs). The presence of polygalacturonase in the model solution lowered PA adsorption from 57.2% to 36.4%. Ruiz-García *et al.* [20] showed that more than 54% of cell wall-bound proanthocyanidins were found within the pectic fraction and that the removal of pectic polysaccharides from cell walls significantly reduced the adsorption of proanthocyanidins. The retention was even lower when CEL was present in the model solution, although the difference with CE was not statistically significant. The presence of this latter enzyme (CE) led to a level of PA retention intermediate between the results of PG and CEL. The lower retention capacity exhibited by CWs when CEL was present may have been due to the effect of cellulose hydrolysis on the release of the inner pectic polymers from within the cell wall [21].

In all of the different experiments, the mDP of the remaining tannins was lower than that of the original tannin, indicating that, in general, there was a preference for the higher molecular mass tannins to be adsorbed as has been observed by several authors [7,22,23]. The effect of the presence of enzymes was to increase the mDP of the remaining tannins compared with the control sample (although the values were still lower than that of the original sample). The presence of PG in the solution gave, as a result, that the remaining PAs in solution presented the most similar mDP to that of the PAs from the control sample (1.90 *vs.* 1.81), meaning that these CWs maintained the most the capacity for interaction with high molecular mass PAs. The PAs remaining in the solution after the interaction of CWs in the presence of CEL or CE presented higher mDP values, closer to that of the original tannin, indicating that the modifications in the CWs due to the presence of these two enzymes limited the interactions with high molecular mass PAs.

The percentage of galloylation mirrored the mDP, decreasing in the PAs remaining after the reaction. The substitution of gallic acid in the PA molecule may increase the number of sites available for the formation of hydrogen bonds between PAs and cell wall components [7], so that those showing a higher galloylation percentage are preferentially adsorbed. The lowest galloylation values in the PAs in solution were obtained in the control sample and when CE was present.

Thus, when the enzymes are present, the retention of PAs by CWs decreased and the CEL activity seemed to be the most significant in this reduction, probably because the degradation of cellulose provides an easier access to the inner pectic polymers and that may favor the loosening of these polysaccharides. From a technological point of view, these results indicate that the use of enzymes capable of modifying the CW structure will limit the PA adsorption and, therefore, facilitate the presence of tannins in solution in must or juice.

However, two questions remain: (a) could the PA somehow inhibit the action of the enzyme (due to the reactivity between proteins and PAs)? and (b) could we rule out the fact that, when PAs are present, there could be a failure of the enzyme to access the polysaccharide network due to steric impediments caused by the PAs occupying the CW surface and, therefore, only partially modifying the CW structure (as suggested by Castro-López *et al.* [13]).

In an attempt to clarify these two questions, in a second experiment, and prior to the addition of the PA, the CWs were immersed in four different model solutions, three containing the different enzymes ((2CW + PG), (2CW + CEL), and (2CW + CE)), and a fourth sample without any enzyme ((2CW)). After 90 min of contact time, the treated CWs were recovered and put in another model solution containing only the PA (Table 2). The supernatant was also recovered, analyzed for its polysaccharide content (Table 3), and also tested for PA interactions by also adding a known amount of PA to this recovered solution (Table 2).

**Table 2:** Concentration and composition of the remaining PAs in solution after the interaction with enzyme pretreated CWs ((2CW)) or with the supernatant solution (S).

Sample	Total mg/L	%Retention *	mDP	%Galloylation
PA	853.47 ± 60.92a	–	2.36 ± 0.01a	11.15 ± 0.02a
(2CW) + PA	420.27 ± 17.41d	50.8	1.90 ± 0.06c	9.43 ± 0.26b
(2CW + PG) + PA	539.46 ± 39.36b	36.8	1.89 ± 0.10c	8.52 ± 0.47c
(2CW + CEL) + PA	578.03 ± 34.59b	32.3	2.06 ± 0.10b	9.68 ± 0.03b
(2CW + CE) + PA	463.74 ± 16.65c	45.7	2.05 ± 0.10b	9.17 ± 0.50b
S + PA	704.59 ± 23.74a	17.5	2.06 ± 0.02a	10.13 ± 0.06a
S(PG) + PA	575.42 ± 19.34b	32.6	2.03 ± 0.16a	9.01 ± 0.06b
S(CEL) + PA	446.70 ± 24.58c	47.7	1.70 ± 0.02b	7.30 ± 0.09c
S(CE) + PA	559.81 ± 41.85b	34.5	2.06 ± 0.01a	8.93 ± 1.33b

* Calculated as the difference between PA concentration in the solution before and after the interaction with CWs. mDP: mean degree of polymerization; (2CW + PA) and S + PA represent the control samples and control supernatant, with no enzyme pre-treatment of CWs; PG: polygalacturonase; CEL: cellulase; CE: commercial enzyme. (2CW). Different letters within the same column and experiment indicate significant differences (*p* < 0.05) according to a Least Significant Difference test.

**Table 3:** Content of uronic acids and total glucose (*mg*/*g* CW) in the supernatants obtained after the interaction of CWs with enzymes, in absence of PAs.

Sample	UA	Glucose
S	6.73 ± 1.88b	0.59 ± 0.06c
S + PG	13.69 ± 2.04a	0.97 ± 0.01b
S + CEL	15.07 ± 0.63a	2.71 ± 0.20a
S + CE	15.70 ± 0.53a	1.15 ± 0.22b

S: supernatants; UA: uronic acids; PG: polygalaturonase; CEL: cellulase; CE: commercial enzyme. Different letters within the same column and experiment indicate significant differences (*p* < 0.05) according to a Least Significant Difference test.

When the CWs from the control sample were used for the interaction study, the retention of PAs was reduced (compared with that measured in experiment 1, the retention values changing from 57.2% to 50.8%), probably due to the action of the ethanol and acid media on the cell wall structure. Some degree of depectination occurred since galacturonic acid was found in the supernatant of the reaction tube (Table 3), which may explain the lower retention of PAs observed in these samples. The studies of Zietsman *et al.* [20] also showed that pectin solubilization and an exposure of the hemicellulose-cellulose framework occurs as a consequence of the fermentation process, due to the apparition of ethanol in the media.

However, when the solution contained PG, no differences were observed between the first and this second experiment, the retention of PAs being similar (36.4% in the first experiment and 36.8% in the second one). Although, when CEL was used, the retention was slightly higher in the second experiment. These results demonstrated the rapidity with which the enzymes acted in both experiments, indicating that, probably, the presence of PAs in the solution did not interfere with the enzymes. The results also demonstrate that the lower retention of CWs treated with PG, compared with control CWs, is due to the loss of polysaccharides, since high quantities of galacturonic acid and total glucose were found in the supernatant when PG was used. Zietsman *et al.* [20] demonstrated that when CWs were treated with pectic enzymes, there was a drastic decrease in the values of homogalacturonan in the CW, indicating that homogalacturonan was degraded and diffused out into the incubation buffer, similar to our findings. The same authors also stated that rammnogalacturonan and xyloglucan were removed, confirming the xyloglucan-pectin association and explaining the large quantities of glucose found in the supernatant.

When CEL was present, the quantities of glucose in the supernatant were maximum (Table 3) but, unexpectedly, the quantities of galacturonic acid were also high. It seems that the elimination of cellulose by cellulase opened up the cell wall structure, rendering access to the inner cell wall spaces and allowing a better access of the acidic ethanolic solution to pectins. In contrast, in the control samples only the outer pectin-rich layers were dissolved, and so only small quantities of galacturonic acid were found in the supernatant. Arnous and Meyer [24] also observed an increase in the values of galacturonic acid in solution after treating grape skin CWs with an enzyme preparation whose main activity was cellulose.

A different case was that of CE. Although the supernatant showed a galacturonic acid and glucose content similar to that of PG treated CWs (similarly to the results found by Arnous and Meyer [24] after combining different enzyme preparations), the CWs from the second experiment retained higher quantities of PAs (36.8% *vs.* 45.7%). Pockets and pores in the polysaccharide gel networks produced by the combination of enzymatic activities in CE [25,26] could favor encapsulation of PAs and, therefore, higher retention. The mDP and %G were similar in experiments one and two.

Less attention has usually been paid to the reactivity of the polysaccharides present in the solution after interaction with CWs. The presence of galacturonic acid and glucose in the solution after acid hydrolysis confirmed the release of pectic polysaccharides as well as hemicellulose and cellulose-derived polysaccharides (Table 3). However, their reactivity towards PAs and their technological significance have not been evaluated.

The results pointed to the substantial reactivity of these polysaccharides with the PAs (Table 2). When PAs were added to the different supernanants, a significant reduction of their concentration was observed. Related to these results, Renard *et al.* [27] described that the presence in solution of soluble polysaccharides, which increase with the presence of enzymes, facilitated the desorption of CW-bound PAs, indicating a high affinity of these soluble polysaccharides for PAs and a competence with the CW polysaccharides. The lowest retention was observed in the control solution, which also presented the lowest quantities of polysaccharides. Greater retention (less quantity of tannins remaining after the interaction) was observed in the supernatant of the CWs treated with PG. Renard *et al.* [28] also found that pectic enzymes were able to efficiently degrade the CW, releasing 78.5% of the galacturonic acid, 85.5% of the glucose, and between 53% and 74% of the minor sugars to the buffer media. Very similar values were observed in the supernatant of the CWs treated with CE, which was to be expected since both presented very similar quantities of polysaccharides. The supernatant of the CWs treated with CEL, which presented the highest quantities of polysaccharides, also showed the highest retention of PAs. From a technological point of view, this may also affect the quantity of PAs in must and juices, since they may not only be adsorbed by CWs, but they may also react with the CW degradation polysaccharides.

**Figure 1:**
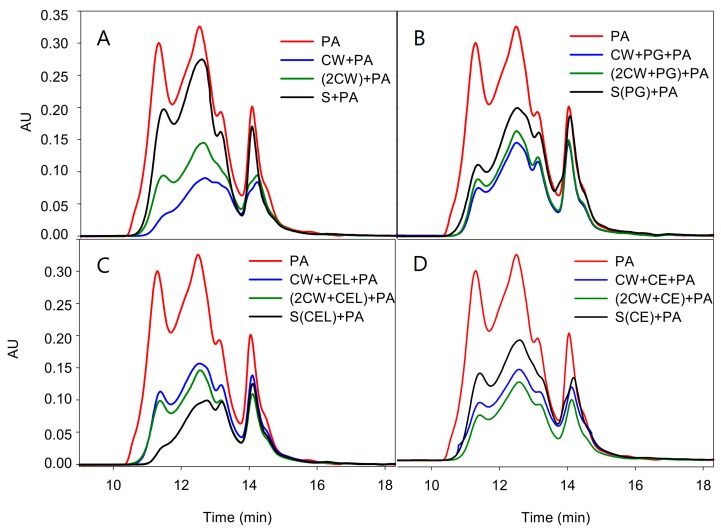
Comparison of the size exclusion chromatogram of the original proanthocyanidin in solution and that for those remaining after (**A**) the first (CW + PA) or second experiment (2CW + PA) as well as those remaining after reacting with the polysaccharides found in the model solution (S + PA); (**B**) the first (W + PG + PA) or second experiment ((2CW + PG) + PA) when polygalacturonase was present, as well as those remaining after reacting with the polysaccharides found in the model solution (S(PG) + PA); (**C**) the first (CW + CEL + PA) or second experiment ((2CW + CEL) + PA) when cellulase was present, as well as those remaining after reacting with the polysaccharides found in the model solution (S(CEL) + PA); and (**D**) the first (CW + CE + PA) or second experiment ((2CW + CE) + PA) when a commercial enzyme was present, as well as those remaining after reacting with the polysaccharides found in the model solution (S(CE) + PA).

It is well known that the information provided by phloroglucinolysis analysis is limited, due to the incomplete conversion of PAs to their constituent subunits and the lack of information on the size distribution of the PA, since only the mean degree of polymerization can be calculated. Therefore, the study was completed by studying the composition of PAs by Size Exclusion Chromatography (SEC) to provide information on the mass distribution of the different PAs studied (Figure 1A–D and Table 4).

Proportionally, the retention of PAs by CWs was greater when observed by SEC, since phloroglucinolysis analysis does not quantify some PAs, mainly oxidized ones, as they are not depolymerized by the phloroglucinolysis reagent and these oxidized PAs present a high affinity for CWs, as observed in other studies [7,9]. The presence of oxidized PAs in grape seeds has previously been reported by other authors [29] and, while the oxidized compounds are not depolymerized by the phloroglucinolysis reaction, they can be analyzed by SEC.

The control CWs retained 70% of the original PAs and, as can be observed and coincident with the data of the phloroglucinolysis analysis, PAs of high molecular mass (the principle of SEC is that molecules of different sizes will elute at different rates, the largest fractions eluting earlier) were the most retained (the differences in the absorbance units of the first eluting fractions between the original PA and those remaining after the adsorption process being large). Also, coincident with the phloroglucinolysis analysis, soaking CWs in an ethanolic model solution decreased their retention capacity (from 70% to 56%) even when enzymes were not present. Additionally, as seen in the phloroglucinolysis analysis, a low retention of PAs by the polysaccharides present in the supernatant was also observed by SEC.

**Table 4:** Total area observed in the size exclusion chromatography analysis for the original PAs and those remaining in solution after the interaction and the percentage of retention calculated.

Sample	Total Área Calculated by SEC	%Retention
PA	0.76	–
CW + PA	0.23	70.2
2CW + PA	0.34	56.1
S + PA	0.57	23.7
CW + PG + PA	0.33	57.3
(2CW + PG) + PA	0.36	53.3
S(PG) + PA	0.49	35.5
CW + CEL + PA	0.38	50.1
(2CW + CEL) + PA	0.33	57.4
S(CEL) + PA	0.24	68.5
CW + CE + PA	0.37	52.6
(2CW + CE) + PA	0.28	64.5
S(CE) + PA	0.48	37.1

When PG was used, the retention of PAs was lower than that observed in the control (as also observed by phloroglucinolysis analysis) but again, proportionally, the results were higher when observed by SEC. However, it is to be noted that the quantities of PAs retained after the second experiment were very similar to those of the first experiment and to the control values of the second experiment itself. It seems that the ethanolic soaking had quite a similar effect to the enzyme. The compounds in the supernatant resulting after treating the CWs with PG had a higher affinity for PAs than that of the control samples, 35.5% of the PAs being retained.

When CEL was assayed, the retention of PAs observed the first experiment was lower than that of the control and PG-treated CWs, confirming that CEL promoted a greater modification of the CWs, which agrees with the high quantity of the liberated polysaccharides found after the reaction of CEL and CWs; this modification reduced their affinity for PAs. The retention values of PAs in the CWs from the second experiment were quite similar to the first one, although slightly higher. However, and contrary to the findings using PG, PA retention was quite high with the polysaccharides present in the supernatant after the treatment with CEL. It seems that PG in the solution promoted an extensive degradation of the liberated pectic polymers, reducing their retention capacity. Probably, degradation of the cellulose network also allowed the release of pectic polymers but the CEL did not promote the extensive degradation of these liberated pectic polymers, explaining the higher affinity of the polysaccharides of this solution towards PAs. This hypothesis needs to be confirmed with more studies and it could have a technological interest since the use of enzymes rich in PG activity could limit the interaction between PAs and CWs and also limit the reactivity of PAs towards the liberated polysaccharides, by degrading them to smaller molecules with less affinity for PAs.

Finally, in the case of commercial enzyme, the values of the retained PAs in the first experiment were similar to those observed with the PG and CEL assays. However, in the second experiment, the cell wall retained more PAs, again confirming the findings of the phloroglucinolysis analysis. As stated before, it is possible that, in this case, the opening of pores increases the retention, even when there was a loss of polysaccharides. The polysaccharides in the supernatant (present in similar quantities to those found after the action of PG) retained a similar percentage of PAs to that retained when PG was used. Again, the more extensive degradation of polysaccharides promoted by the pectic enzymes present in the CE reduced their affinity for PAs.

## 3. Materials and Methods

### 3.1. Chemicals

Chromatographic solvents were of high-performance liquid chromatography (HPLC) grade, and chemicals were of analytical reagent grade. Acetonitrile, acetone, chloroform, methanol, ethanol, formic acid, and trifluoracetic acid were from Merck (Darmstadt, Germany). The phloroglucinol reagent and tris-HCl equilibrated phenol pH 6.7 were sourced from Sigma Aldrich (St. Louis, MO, USA). Sodium acetate was from J.T. Baker (Deventer, The Netherlands). The (+)-catechin, (−)-epicatechin, (−)-epicatechin gallate and (−)-epigallocatechin standards were obtained from Extrasynthese (Genay, France). Galacturonic acid was from Sigma (St. Louis, MO, USA) and Bovine serum albumin (BSA) fraction V from J.T. Baker (Deventer, Holland). For glucose determination, an enzymatic analysis kit from R-biopharm (Darmstadt, Germany) was used.

### 3.2. Instrumentation

The HPLC apparatus was a Waters 2695 (Waters, Milford, MA, USA) equipped with a Syrahstem autosampler, and a Waters 2996 photodiode array detector (Waters, Milford, MA, USA).

### 3.3. Cell Wall Material

Cell walls (CW) were isolated from fresh skins of *Vitis vinifera* L. cv. Monastrell following the method of De Vries *et al.* [30] described by Castro-López *et al.* [13]. Briefly, skins were extracted in 70% *v*/*v* acetone to remove proanthocyanidins and, then, residues were washed in an additional 70% *v*/*v* acetone, followed by Milli-Q water and were then homogenized under liquid nitrogen. Acetone-insoluble residues from skins were treated with tris-HCl equilibrated phenol pH 6.7, and then washed with 80% *v/v* methanol and with acetone to remove the phenol. Samples were then shaken for 30 min in 1:1 *v:v* methanol/chloroform and washed with methanol and with acetone. The insoluble residue was then lyophilized and the recovered CWs were manually ground to a fine particle size with a mortar and pestle before freezing at −20 °C until use.

### 3.4. Analysis of Polysaccharide Composition of the Supernatants

Uronic acids were determined in the solution by the colorimetric 3,5-dimethylphenol assay after pretreatment (30 °C, 1 h) with aq. 72% sulphuric acid followed by hydrolysis with 1 M sulphuric acid (100 °C, 3 h). Pure galacturonic acid was used as a standard.

The total glucose was determined using a kit for glucose enzymatic analysis from R-biopharm (Darmstadt, Germany) after pretreatment (30 °C, 1 h) with aqueous 72% sulphuric acid, followed by hydrolysis with 1 M sulphuric acid (100 °C, 3 h).

### 3.5. Proanthocyanidins Used in the Interaction Studies

A seed-derived commercial tannin (TanReactive, Agrovin, Alcazar de San Juan, Spain) with a purity of 42.65%, a degree of polymerization of 2.36 and galloylation percentage of 11.2% was used. The purity and the concentration and composition of the commercial tannin were previously determined following the method of Ribereau-Gayon *et al.* [31] the samples were depolymerized by phloroglucinolysis.

### 3.6. Binding Reactions between Tannins and Cell Wall Material in the Presence and Absence of Enzyme

For the first experiment, CW samples were combined in tubes of 3 mL with the enological tannin previously dissolved in a model solution (12% ethanol and pH 3.6 adjusted with trifluoroacetic acid) to obtain a final concentration in the medium of 2 g/L of PAs and 13 mg/mL of CWs. This experiment was conducted in triplicate and with four different sets of samples: a triplicate set containing polygalacturonase (EC 3.2.1.15, *ca.* 600 units/g of solid, provided by Sigma, St. Louis, MO, USA), another set containing cellulase (EC 3.2.1.4, 1.92 units/mg of solid, provided by Sigma, St. Louis, MO, USA, both enzymes at a dose of 100 mg/L), another set containing a commercial enzyme supplied by Agrovin S.A. (Alcazar de San Juan, Spain, the CE samples) at a dose of 15 mg/L, and a control set of samples, with no enzyme addition. The samples were shaken at 300 rpm in an orbital shaker at room temperature for 90 min. A blank without CW was also carried out. After the binding reaction, samples were centrifuged at 13,000 rpm and the supernatant was then dried under vacuum at 35 °C. The recovered tannin was dissolved in 250 μL of methanol before analysis by phloroglucinolysis and size exclusion chromatography (SEC).

For the second experiment, another four sets of CW samples were suspended in the model solution, now each set containing polygalacturonase (PG), cellulase (CEL) or the commercial enzyme (CE). Another set contained no enzyme added (control samples). Each set was prepared in sixtuplicate. The samples were shaken at 300 rpm in an orbital shaker at room temperature for 90 min, after which they were centrifuged at 13,000. The supernatants were transferred to new tubes, three of which were used for the interaction reactions with the PA and the other to analyze the polysaccharide content. The CWs found in the precipitate were re-dissolved in a fresh model solution that only contained the PA sample. The different samples were treated under the same conditions described above to monitor the adsorption of tannins. After the binding reaction, the same procedure described above was applied.

### 3.7. Analysis of Tannins by Phloroglucinolysis

The tannin content of the control sample (2 g/L) and the tannins remaining in solution after interaction with the CWs or with the supernatants obtained after the enzymatic treatment of CWs were analyzed using the phloroglucinol reagent, following the method proposed by [32] with some modifications [13]. The methanolic extract was left to react with the phloroglucinolysis reagent (1:1) (a solution of 0.2 M HCl in methanol, containing 100 g/L phloroglucinol and 20 g/L ascorbic acid) in a water bath for 20 min at 50 °C and then combined with two volumes of 200 mM aqueous sodium acetate to stop the reaction.

HPLC analysis followed the conditions described by Busse-Valverde *et al.* [33]. Water/formic acid (98:2, *v*/*v*) was used as solvent A and acetonitrile/water/formic acid (80:18:2 *v*/*v*/*v*) as solvent B. The rate flow was 0.8 mL/min flow and the oven temperature 30 °C. Elution began with a linear gradient from 0 to 10% B in 25 min, gradient from 10% to 15% in 20 min and gradient from 15% to 22% in 15 min, followed by washing and re-equilibration of the column.

Proanthocyanidin cleavage products were estimated using their response factors at 280 nm relative to (+)-catechin, which was used as the quantitative standard. The recovery by mass of the total proanthocyanidin content, the apparent mean degree of polymerization (mDP) and the percentage of each constitutive unit were determined.

### 3.8. Analysis of Tannins by Size Exclusion Chromatography

An adaptation of the method described by Kennedy and Taylor [34] was used for size exclusion chromatography (SEC) and can be found in Bautista-Ortín *et al.* [7]. Briefly, this method utilized two PLgel columns (300 × 7.5 mm, 5 μm, 500 (effective molecular mass range of up to 4000 using polystyrene standards)) by 100 Å (effective molecular mass range of 500–30,000 using polystyrene standards). These columns were connected in series (Polymer Labs, Amherst, MA, USA), and an isocratic method was used, the mobile phase consisting of N,N-dimethylformamide containing 1% glacial acetic acid, 5% water and 0.15 M lithium chloride. The sample injection volume was 10 μL, the flow rate 1 mL/min and oven temperature 60 °C. The elution was monitored at 280 nm.

### 3.9. Statistical Analysis

All the statistical analyses were made using Statgraphics Centurion XVI (Statpoint Technologies Inc., Warrenton, VA, USA).

## 4. Conclusions

Our studies have shown that the presence of the different enzymes in the model solution lowered, in all cases, the retention of the PA by the CWs and that the reactivity of the enzymes was not affected by the PA present in the reaction media. Cellulase was the enzyme that most affected the CW-PA retention, perhaps because hydrolysis of the cellulose microfibrils influences the release of the inner pectic polymers from within the cell wall, which may favor the loosening of these polysaccharides. This hypothesis was supported by the fact that high quantities of galacturonic acid and total glucose were found in the model solution recovered after the CWs were treated with the enzymes, especially when cellulase was used. However, these liberated polysaccharides also presented a high affinity for the PAs and may reduce the PA content in the solution.

From a technological point of view, these results show that the CW structure may be manipulated by deconstructing enzymes that will limit the adsorption of PAs and therefore increase the presence of tannins in musts or juices. However, and given that our results also proved that the liberated polysaccharides can also interact with the PAs in the solution, the studies must keep on trying to find the best enzyme or combination of enzymes that would limit both the CW-PA interactions and the PA interactions with the liberated polysaccharides.

## Abbreviations

PA: Proanthocyanidin; CW: Cell walls; PG: Polygalacturonase; CEL: Cellulase; CE: Commercial maceration enzyme; SEC: Size exclusion chromatography.

## References

[1] McManus J., Davis K., Beart J., Gaffney S., Lilley T., Haslam E. (1985). The association of proteins with polyphenols. J. Chem. Soc. Perkin III.

[2] Cai Y., Gaffney S., Lilley T., Haslam E., Hemingway R. (1989). Carbohydrate-polyphenol complexation. Chemistry and Significance of Condensed Tannins.

[3] Riou V., Vernhet A., Doco T., Moutounet M. (2002). Aggregation of grape seed tannins in model wine-effect of wine polysaccharides. Food Hydrocoll..

[4] Padayachee A., Netzel G., Netzel M., Day L., Zabaras D., Mikkelsen D., Gidley M. (2012). Binding of polyphenols to plant cell wall analogues. Part I. Anthocyanins Food Chem..

[5] Martínez-Hernández A. (2015). Interacción de las proantocianidinas con las paredes celulares. Influencia de la concentracion de antocianos. Master’s Thesis.

[6] Bindon K., Smith P., Holt H., Kennedy J. (2010). Interaction between grape-derived proanthocyanidins and cell wall material. 2. Implications for vinificatio. J. Agric. Food Chem..

[7] Bautista-Ortin A.B., Cano-Lechuga M., Ruiz-García Y., Gómez-Plaza E. (2014). Interactions between grape skin cell wall material and commercial enological tannins. Practical implications Food Chem..

[8] Le Bourvellec C., Le Quere J.M., Renard C.M.G.C. (2007). Impact of Noncovalent Interactions between apple condensed tannins and cell walls on their transfer from fruit to juice: Studies in model suspensions and application. J. Agric. Food Chem..

[9] Le Bourvellec C., Guyot S., Renard C.M. (2009). Interactions between apple (*Malus x domestica Borkh.*) polyphenols and cell walls modulate the extractability of polysaccharides. Carbohydr. Polym..

[10] Le Bourvellec C., Gouble B., Bureau S., Loonis M., Ple Y., Renard C.M. (2013). Pink discoloration of canned pears: Role of procyanidin chemical depolymerization and procyanidin/cell wall interactions. J. Agric. Food Chem..

[11] Romero-Cascales I., Fernández-Fernández J.I., Ros-García J.M., López-Roca J.M., Gómez-Plaza E. (2008). Characterisation of the main enzymatic activities present in six commercial macerating enzymes and their effects on extracting colour during winemaking of Monastrell grapes. Int. J. Food Sci. Technol..

[12] Bautista-Ortín A.B., Martínez-Cutillas A., Ros-García J.M., López-Roca J.M., Gómez-Plaza E. (2005). Improving colour extraction and stability in red wines: The use of maceration enzymes and enological tannins. Int. J. Food Sci. Technol..

[13] Castro-Lopez L.R., Gómez-Plaza E., Ortega-Regules A., Lozada D., Bautista-Ortin A.B. (2016). Role of cell wall deconstructing enzymes in the proanthocyanidin—cell wall adsorption—desorption phenomena. Food Chem..

[14] Bindon K., Smith P. (2013). Comparison of the affinity and selectivity of insoluble fibres and commercial proteins for wine proanthocyanidins. Food Chem..

[15] Guerrero R., Smith P., Bindon K. (2013). Application of insoluble fibers in the fining of wine phenolics. J. Agric. Food Chem..

[16] Bautista-Ortín A.B., Ruiz-García Y., Marín F., Molero N., Apolinar-Valiente R., Gómez-Plaza E. (2015). Remarkable proanthocyanidin adsorption properties of Monastrell pomace cell wall material highlight its potential use as an alternative fining agent in red wine production. J. Agric. Food Chem..

[17] Zietsman J.J. (2015). Investigating Grape Berry Cell Wall Deconstruction by Hydrolytic Enzymes.

[18] Pabst M., Fischl R.M., Brecker L., Morelle W., Fauland A., Köfeler H., Altman F., Leonard R. (2013). Rhamnogalacturonan II structure shows variation in the side chains monosaccharide composition and methylation status within and across different plant species. Plant J..

[19] Agrovin. http://www.agrovin.com/agrv/pdf/enologia/enzimas/es/Enozym_VINTAGE_es.pdf.

[20] Ruiz-García Y., Smith P., Bindon K. (2014). Selective extraction of polysaccharide affects the adsorption of proanthocyanidin by grape cell walls. Carbohydr. Polym..

[21] Zietsman J.J., Moore J., Fangel J., Willats W., Vivier M. (2015). Profiling the Hydrolysis of isolated grape berry skin cell walls by purified enzymes. J. Agric. Food Chem..

[22] Haslam E. (1998). Molecular recognition. Phenols and polyphenols. Practical Polyphenolics. From Structure to Molecular Recognition and Physiological Action.

[23] Bindon K., Madani S., Pendleton P., Smith P., Kennedy J. (2014). Factors affecting skin tannin extractability in ripening grapes. J. Agric. Food Chem..

[24] Arnous A., Meyer A. (2010). Discriminated release of phenolic substances from red wine grape skins (*Vitis vinifera* L.) by multicomponent enzymes treatment. Biochem. Eng. J..

[25] Le Bourvellec C., Guyot S., Renard C.M., Abbal P. (2004). Non-covalent interaction between procyanidins and apple cell wall material: Part I. Effect of some environmental parameters. Biochim. Biophys. Acta.

[26] Le Bourvellec C., Bouchet B., Renard C.M. (2005). Non-covalent interaction between procyanidins and apple cell wall material. Part III: Study on model polysaccharides. Biochim. Biophys. Acta.

[27] Renard C.M., Baron A., Guyot S., Drilleau J.F. (2001). Interactions between apple cell walls and native apple polyphenols: quantification and some consequences. Int. J. Biol. Macromol..

[28] Renard C.M. (2005). Effects of conventional boiling on the polyphenols and cell walls of pears. J. Sci. Food Agric..

[29] Passos C.P., Cardoso S.M., Domingues M.R., Domingues P., Silva C.M., Coimbra M.A. (2007). Evidence for galloylated type-A procyanidins in grape seeds. Food Chem..

[30] De Vries J., Voragen A.G.L., Rombouts M., Pilnik W. (1981). Extraction and purification of pectins from alcohol insoluble solids from ripe and unripe apples. Carbohydr. Polym..

[31] Ribéreau Gayon P., Glories Y., Maujean A., Dubourdieu D. (1998). Traité d‘Oenologie.2. Chimie du vin.

[32] Pastor del Rio J.L., Kennedy J.A. (2006). Development of proanthocyanidins in *Vitis vinifera* L. cv. Pinot noir grapes and extraction into wine. Am. J. Enol. Vitic..

[33] Busse-Valverde N., Gómez-Plaza E., López-Roca J.M., Gil-Muñoz R., Fernández-Fernández J.I., Bautista-Ortín A.B. (2010). Effect of different enological practices on skin and seed proanthocyanidins in three varietal wines. J. Agric. Food Chem..

[34] Kennedy J.A., Taylor A.W. (2003). Analysis of proanthocyanidins by high performance gel permeation chromatography. J. Chromatogr..

